# Peripapillary vessel density measurement of quadrant and clock-hour sectors in primary angle closure glaucoma using optical coherence tomography angiography

**DOI:** 10.1186/s12886-021-02093-0

**Published:** 2021-09-09

**Authors:** Yongdong Lin, Shirong Chen, Mingzhi Zhang

**Affiliations:** Joint Shantou International Eye Center of Shantou University and The Chinese University of Hong Kong, Dong xia Road, Shantou, Guangdong Province People’s Republic of China

**Keywords:** Optical coherence tomography angiography, Peripapillary vessel density, Primary angle closure glaucoma

## Abstract

**Background:**

The purpose of this study was to investigate diagnostic ability of peripapillary vessel density of primary angle closure glaucoma (PACG) eyes in quadrant and clock-hour sectors by optical coherence tomography angiography (OCTA).

**Methods:**

This was a cross-sectional study on forty-one PACG patients (41eyes) and twenty-seven healthy subjects (27 eyes). All subjects underwent OCTA (DRI OCT Triton; Topcon Corporation, Tokyo, Japan) and peripapillary retinal nerve fiber layer (RNFL) thickness imaging with swept-source optical coherence tomography (OCT). The peripapillary vessel density of quadrant and clock-hour sectors was quantified by imageJ software. The diagnostic capability of OCTA and OCT parameters was evaluated by the areas under the receiver operating characteristics curves (AUCs). Pearson correlation analysis or Spearman correlation test was used to evaluate the correlation between vessel density parameters and related factors.

**Results:**

Compared with the control group, the peripapillary vessel density of glaucomatous group was lower to different degrees in the four quadrants and each clock-hour sectors, and vessel density reduced most at 7 o’clock. The difference between the diagnostic ability of peripapillary vessel density and peripapillary RNFL thickness was not statistically significant, except 4 o’clock and inferior quadrant. The inferior quadrant peripapillary vessel density had the best diagnostic value (AUC0.969), followed by the 7 o’clock vessel density (AUC0.964), average vessel density (AUC0.939) and the 7 o’clock RNFL thickness (AUC0.919). The average peripapillary vessel density was correlated with average RNFL and visual field (VF) mean deviation (*P < *0.001).

**Conclusions:**

In PACG, the diagnostic ability of the peripapillary vessel density is equivalent to the peripapillary RNFL thickness. Understanding spatial characteristics of the peripapillary vessel density in PACG may be helpful for clinical diagnosis and monitoring the progress of diseases.

## Background

There was increasing evidence showing that vascular factors were closely related to the pathogenesis of glaucoma [1]. In the past, there was a lack of effective non-invasive instruments to study the mechanism of vascular factors in glaucoma [2–5]. Recently, research on glaucoma by optical coherence tomography angiography (OCTA) showed that the peripapillary vessel density was lower, which exhibited a good diagnostic ability for glaucoma [5, 6]. Previous studies used OCTA to reveal the spatial characteristics of peripapillary vessel density in glaucoma mainly on average and quadrants, which, however, were limited by the underutilization of the data from each clock-hour sectors [7, 8]. In addition, it has been proven that the changes of neuroretinal rim in the eyes of glaucoma patients can be diffuse or local. Therefore, understanding the clock-hour spatial characteristics of peripapillary vessel density may help us to further understand the vascular mechanism of glaucoma and improve the diagnostic ability for glaucoma. As far as we know, there have not been any publication that investigates the peripapillary clock-hour vessel density of primary angle closure glaucoma (PACG).

We know little about whether optic disc capillary atrophy in glaucoma eye is diffuse or localized. Shin et al. [9] studied peripapillary vessel density in eyes of normal-tension glaucoma (NTG) at clock-hour sectors using OCTA. They reported that the superficial and deep vessel density of glaucoma at 7 and 11 o’clock positions decreased mostly compared with healthy eyes. The diagnostic ability of 7 o’clock position was the highest. The mechanism of PACG is different from NTG and primary open angle glaucoma (POAG) [10]. We do not know whether the peripapillary vessel density is the same for PACG.

Therefore, the purpose of this study was to study the quadrant and clock-hour spatial characteristics of peripapillary vessel density in PACG, and to assess the diagnostic ability of vessel density.

## Materials and methods

### Study subjects

We told each subject the content of the study. Their willingness to participate was recorded and they voluntarily signed the consent form approved by the institutional ethics committee. PACG patient and healthy person were selected from October 2018 to October 2019 in Joint Shantou International Eye Center. All subjects underwent a detailed medical history, best corrected visual acuity ( BCVA), intraocular pressure (IOP) measurement, axial length measurement by OA-2000 (Tomey GmbH, Nagoya, Japan), fundus examination, swept-source optical coherence tomography (OCT), and OCTA (DRI OCT Triton, TOPCON) examination. We utilized the same DRI OCT instrument to perform OCT and OCTA scans. Systolic blood pressure (SBP) and diastolic blood pressure (DBP) of the subjects were measured before OCTA was measured. The ocular perfusion pressure (OPP) was calculated as the following formula:

$$OPP=2/3\left(\mathrm{DBP}+0.42\left(\mathrm{SBP}-\mathrm{DBP}\right)\right)-\mathrm{IOP}$$ [11]

Glaucoma patients also underwent visual field (VF) examination by the static automated white-on-white threshold 24 − 2 SITA standard strategy (Humphrey Field Analyzer II; Carl Zeiss Meditec). Our study only included reliable VF examination results (i.e., false-negative errors < 15 %, false-positive errors < 15 %, and fixation loss < 20 %).

The diagnostic criteria for PACG were as follows [12]: 1.the presence of an occluded angle defined as an angle in which > 270° of the posterior trabecular meshwork cannot be seen, verified by gonioscopy. 2. IOP of more than 21 mmHg at the time of diagnosis. 3. optic disc and RNFL damage due to glaucoma. Inclusion criteria for healthy eyes:1. age: > 18 years old. 2. normal anterior chamber ,open-angle and normal fundus in clinical examination by experts. 3. intraocular pressure ≤ 21mmmhg. 4. no family history of glaucoma. Exclusion criteria for all participants were: (1) diopter ≥ 6.0 D (sphere) and or 3.0 D (cylinder). (2) history of other ocular disease, eye surgeries or ocular laser surgery. (3) poor OCT or OCTA image quality score (less than 40 points).

### OCTA imaging acquisition and processing

All subjects were examined by 4.5 × 4.5mm OCTA optic disc scans (DRI OCT Triton; Topcon Corporation, Tokyo, Japan). Images were analyzed by an A-scan rate of 100,000 scans per second, wavelength-scanning light centered on 1,050nm, and in-depth digital resolution of 2.6mm [13]. We used OCT angiography ratio analyses (OCTARA) of the device to obtain the images. The system automatically divided the optic disc into four layers, and the selected layer was Nerve Head layer. Because Nerve Head layer is defined as 130 μm below the internal limiting membrane which includes the whole retinal capillary network anatomically. Peripapillary vessel density measurement was calculated in the Nerve Head layer.

**Fig. 1:**
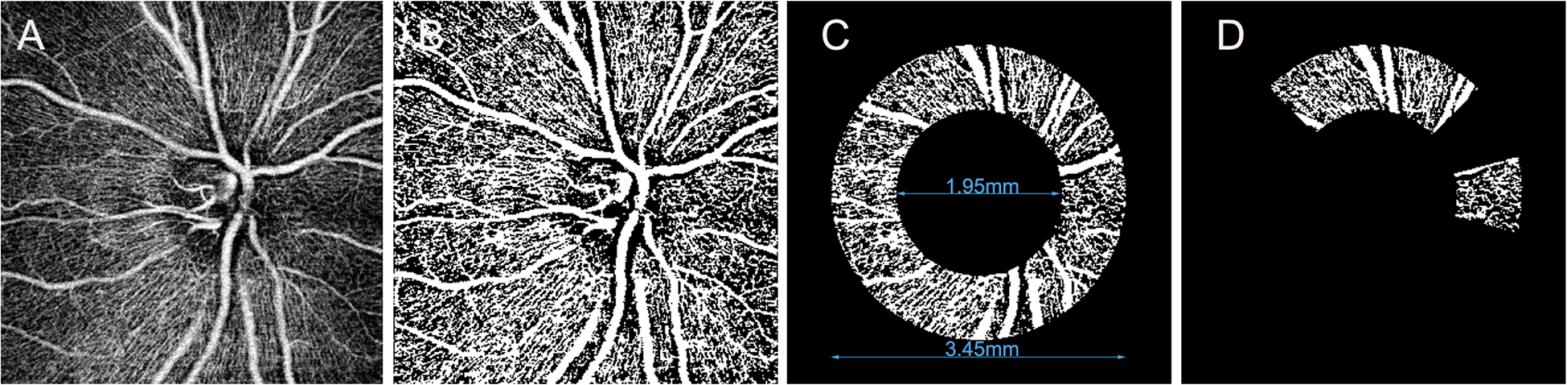
OCT-A image processing steps. **A** Image imported into the Image J software and noise reduction processing. **B** Binary image after local adaptive thresholding. **C** Annular region of interest centered at the optic nerve head. **D** Obtaining vessel density values of each quadrant and clock-hour sector

 We reviewed and filtered images quality after each scan. Images with significant motion artifacts, poor signal strength (signal strength index < 40), or poor image clarity were discarded. We used Image J software (National Institutes of Health, Bethesda, MD) to process OCTA images. First, we applied a non-local means denoising filter to reduce background noise of grayscale images [14]. Then, we performed the Phansalkar as adaptive local thresholding methods [15]. This method was used for binarization algorisms in OCTA images to obtain vascular signals as a white region and digitize this area. The vessel density value was defined as a proportion of vessel signal in area of interest. The reproducibility of this vessel density analysis method was proven by Shoji et al [16]. The peripapillary region was 750mm -wide annular region of interest centered on the optic disc, with an inner diameter of 1.95 cm and an outer diameter of 3.45 cm [17]. The peripapillary region was divided into 4 equal quadrants, namely superior, inferior, temporal and nasal quadrants. The peripapillary region was also divided into 12 clock-hour sectors. OCTA image processing steps were shown in Fig. 1.

### OCT imaging acquisition

All participants were examined with swept-source OCT (DRI OCT Triton; Topcon Corporation, Tokyo, Japan), using an optic disc scan (6 mm x 6 mm, 512 A-scans×256 B-scans). The thickness of the peripapillary retinal nerve fiber layer (RNFL) was obtained from a circle with a diameter of 3.4 mm centered on the optic disc. The RNFL thicknesses in 4 quadrants and 12 clock-hour were obtained by using the built-in automated segmentation algorithm of the system. All subjects underwent OCT and OCTA scanning on the same day.

### Statistical analyses

Data analysis was carried out using commercially available software (SPSS ver.13.0; SPSS Inc, Chicago, Illinois, USA) and MedCalc (ver.15.2.2, Mariakerke, Belgium). We used mean with 95 % credible intervals for continuous variables and the Shapiro-Wilk test to evaluate the normal distribution of continuous variables. Independent t test and Mann–Whitney test were used to observe the differences between the PACG eyes and healthy eyes. We calculated and compared the areas under the receiver operating characteristics curves (AUCs) to evaluate the diagnostic ability for glaucoma of average, quadrant and clock-hour vessel density, as well as peripapillary RNFL thickness. Pearson correlation analysis or Spearman correlation test was used to evaluate the correlation between vessel density parameters and related factors. A *P*-value of < 0.05 was taken to be of statistical significance.

## Results

Forty-eight PACG patients(48 eyes)and 33 healthy people (33 eyes) were enrolled in this study. Seven eyes of PACG patients and 6 eyes of healthy subjects were excluded due to poor OCTA image quality. Therefore, 41 PACG patients (41 eyes) and 27 healthy subjects (27 eyes) were enrolled. Demographics and clinical characteristics of study subjects are listed in Table 1. The axial length of PACG eyes was significantly different from that of healthy eyes (*P < *0.001), and there were no significant differences in age, sex, intraocular pressure at examination and ocular perfusion pressure (*P > *0.05). The VF mean deviation in PACG eyes was − 19.4db.

**Table 1:** Demographic and clinical characteristics of the study population

	Healthy eyes (***n*** = 27)	PACG eyes (***n*** = 41)	***P*** value
Gender (male/Female)	7:20	17:24	0.190*
Age (year)	61.9(58.9-65.9)	62.7(60.1-65.3)	0.699
SBP (mmHg)	137.6(132.7-142.4)	136.0(130.0-142.4)	0.782^†^
DBP (mmHg)	84.8(82.0-87.6)	80.5(77.3-83.7)	0.063^†^
OPP (mmHg)	57.4(54.7-60.1)	54.1(51.0-57.2)	0.155^†^
BCVA (log MAR)	0.6(0.5-0.7)	0.5(0.4-0.6)	0.087
IOP at imaging (mmHg)	13.9(12.7-15.0)	15.1(14.0-16.4)	0.089^†^
Al (mm)	23.6(23.2-23.9)	22.5(22.1-22.8)	<0.001^†^
VF MD (dB)	-	-19.4(-22.4--16.3)	-

Unless otherwise illustrated, the comparison was made by using independent sample t test

*PACG* primary angle closure glaucoma, *SBP* systolic blood pressure, *DBP* diastolic blood pressure, *OPP* ocular perfusion pressure, *BCVA* best-corrected visual acuity, *IOP* intraocular pressure, *AL* axial length, *VF MD* visual field mean deviation

*The comparison was performed by using the chi-square test

^†^The comparison was performed by using the Mann–Whitney test

**Fig. 2:**
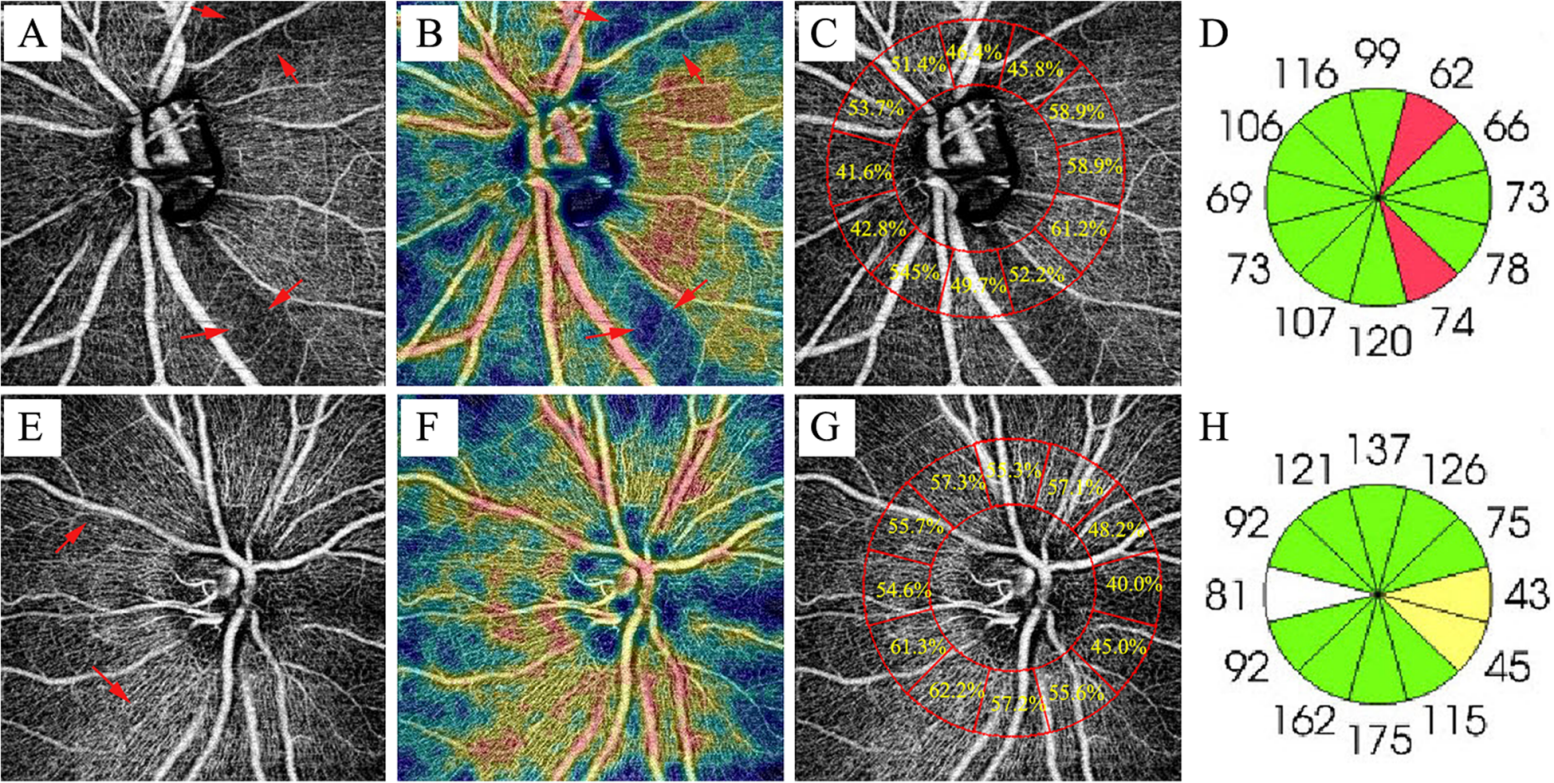
Example of PACG eye with decreased infratemporal and supratemporal vessel density of optic disc. The infratemporal and supratemporal peripapillary vessel density of glaucoma decreased and the peripapillary retinal nerve fiber layer in corresponding areas atrophied. (first row) On the contrary, these areas were very high in healthy eyes. (second row) (**A**) Nerve Head scan of glaucoma eye and (**B**) Corresponding color-coded vessel density map of the Nerve Head layer (**C**) Vessel density value at clock-hour region of glaucoma eye (**D**) Peripapillary retinal nerve fiber layer thickness at clock-hour region of glaucoma eye (**E**) Nerve Head scan of healthy eye and (**F**) Corresponding color-coded vessel density map of the Nerve Head layer (**G**) Vessel density value at clock-hour region of healthy eye (**H**) Peripapillary retinal nerve fiber layer thickness at clock-hour region of healthy eye

Table 2 shows data of the peripapillary vessel density and RNFL thickness on the mean average, quadrant, and clock-hour regions. Compared with the control group, the peripapillary vessel density of glaucoma in all measured sectors was lower in different degrees and the difference was significant (*P* < 0.05), with 7 o’clock decreased most. Except for 3 o’clock (*P* > 0.05), peripapillary RNFL thickness in all measured sectors showed significant difference between the glaucoma group and control group (*P *< 0.05). Figure 2 shows a decrease in infratemporal and supratemporal peripapillary vessel density sectors of glaucoma and atrophy of RNFL thickness in corresponding regions.

**Table 2:** Peripapillary retinal nerve fiber layer thickness and peripapillary vessel density in healthy and glaucomatous eyes

Parametres	RNFL thickness			Peripapillary vessel density		
	Healthy eyes (*n* = 27)	PACG eyes (*n* = 41)	*P* value	Healthy eyes (*n* = 27)	PACG eyes (*n* = 41)	*P* value
Average	113.0(109.2-116.8)	77.1(68.9-853)	<0.001*	54.8(53.8-55.8)	45.3(43.6-46.9)	<0.001*
Temporal	85.4(81.4-89.5)	63.8(58.0-69.5)	<0.001*	54.8(52.7-56.9)	46.6(44.4-48.7)	<0.001
Superior	142.7(135.1-150.4)	92.7(81.0-104.5)	<0.001*	57.3(56.3-58.4)	47.6(45.6-49.6)	<0.001*
Nasal	76.7(71.0-82.5)	63.9(57.0-70.7)	0.009	49.0(46.8-51.1)	41.6(39.3-43.9)	<0.001
Inferior	147.6(142.5-152.7)	86.5(73.9-99.2)	<0.001*	58.3(57.1-59.4)	45.4(43.5-47.2)	<0.001*
9	72.1(69.0-75.2)	60.3(55.9-64.7)	0.001*	50.3(47.5-53.2)	45.9(43.8-47.9)	0.010
10	100.1(94.6-105.7)	68.9(60.6-77.1)	<0.001*	58.4(56.5-60.3)	48.9(46.4-51.5)	<0.001*
11	148.3(138.5-158.2)	90.4(78.6-102.3)	<0.001*	59.2(57.8-60.7)	46.8(44.0-49.7)	<0.001*
12	144.8(132.6-157.0)	99.6(85.9-113.4)	<0.001	56.9(55.5-58.3)	49.4(47.1-51.7)	<0.001*
1	135.1(125.8-144.4)	87.9(76.1-99.8)	<0.001*	55.9(54.4-57.4)	46.7(44.6-48.8)	<0.001*
2	90.9(82.1-99.6)	72.8(64.1-81.4)	0.006	51.0(48.6-53.3)	43.7(41.1-46.2)	0.001*
3	66.7(61.0-72.5)	58.9(52.5-65.2)	0.084	46.7(43.9-49.5)	40.2(37.6-42.9)	0.001
4	72.5(67.8-77.3)	58.6(51.0-66.1)	0.011*	49.5(47.5-51.5)	40.8(38.1-43.5)	<0.001*
5	123.1(116.7-129.5)	83.1(71.2-95.0)	<0.001*	55.7(53.8-57.5)	43.6(41.2-45.9)	<0.001*
6	157.9(150.0-165.9)	98.2(83.3-113.0)	<0.001*	58.7(57.1-60.3)	48.2(46.1-50.4)	<0.001*
7	161.3(151.1-171.5)	81.9(68.2-95.6)	<0.001*	60.4(58.5-62.3)	44.3(41.8-46.7)	<0.001*
8	82.7(76.0-89.4)	61.8(55.2-68.3)	<0.001*	55.2(52.6-57.7)	45.1(42.6-47.6)	<0.001

Unless otherwise illustrated, the comparison was made by using independent sample t test

*RNFL* retinal nerve fiber layer, *PACG* primary angle closure glaucoma

*The comparison was performed by using the Mann–Whitney test

The AUC of peripapillary vessel density was between0.676 (in 9 o’clock sector) and 0.969 (in the inferior quadrant). The AUC of RNFL thickness was between 0.620 (in 3 o’clock sector) and 0.919 (in 7 o’clock sector). Inferior quadrant vessel density had the highest diagnostic ability (AUC0.969), followed by the 7 clock-hour peripapillary vessel density (AUC0.964), average vessel density (AUC0.939) and the 7 o’clock RNFL thickness (AUC0.919). Except 4 o’clock and inferior quadrant, there was no statistical difference between the diagnostic ability of peripapillary vessel density and RNFL. The diagnostic ability of 4 o’clock and inferior quadrant vessel density was higher than that of RNFL thickness (*P* = 0.027, *P* = 0.030) (Table 3).

**Table 3:** Areas under the receiver operating characteristics curves comparison for glaucomatous discrimination ability between Peripapillary vessel density and RNFL thickness

Parametres	Control and glaucomatous eyes				*P* value
	Peripapillary vessel density		RNFL thickness		
Optic disc	AUC	95%CI	AUC	95%CI	
Average	0.939	0.853-0.982	0.891	0.791-0.983	0.193
Temporal	0.830	0.719-0.910	0.833	0.723-0.912	0.953
Superior	0.918	0.825-0.971	0.863	0.758-0.934	0.248
Nasal	0.797	0.682-0.885	0.681	0.557-0.789	0.050
Inferior	0.969	0.896-0.996	0.898	0.801-0.958	0.030
9	0.676	0.551-0.784	0.755	0.635-0.851	0.273
10	0.838	0.729-0.916	0.840	0.731-0.917	0.980
11	0.896	0.798-0.957	0.892	0.793-0.954	0.922
12	0.824	0.712-0.906	0.799	0.685-0.887	0.661
1	0.864	0.760-0.935	0.853	0.746-0.927	0.827
2	0.776	0.658-0.868	0.703	0.580-0.808	0.173
3	0.722	0.600-0.824	0.620	0.494-0.735	0.139
4	0.818	0.706-0.901	0.684	0.560-0.791	0.027
5	0.907	0.812-0.964	0.842	0.733-0.919	0.109
6	0.906	0.810-0.963	0.852	0.745-0.926	0.218
7	0.964	0.889-0.994	0.919	0.827-0.971	0.148
8	0.838	0.729-0.916	0.786	0.670-0.876	0.371

*RNFL* retinal nerve fiber layerm, *AUCs* areas under the receiver operating characteristics curves, *CI* confidence interval

Average RNFL thickness and VF mean deviation were correlated with average peripapillary vessel density. The correlation between average peripapillary vessel density and average RNFL thickness, VF mean deviation were stronger than any other variables (Table 4).

**Table 4:** The correlation between peripapillary vessel density and glaucomatous related factors

	Peripapillary vessel density	
	r	***P***
Age (year)	-0.213	0.181
Sex	0.092	0.569
IOP (mmHg)	-0.178	0.264*
Mean OPP (mmHg)	0.098	0.544
RNFL thickness (μm)	0.753	<0.001
VF MD (dB)	0.691	<0.001*

Unless otherwise illustrated, correlation analysis was made by using Pearson correlation test

*IOP* intraocular pressure, *OPP* ocular perfusion pressure, *RNFL* retinal nerve fiber layer, *VF MD* visual field mean deviation

^*^Correlation analysis was made by using Spearman correlation test

## Discussion

There are limited reports on the peripapillary vessel density in PACG. To the best of our knowledge, this is the first study to report the peripapillary vessel density in clock-hour sector in PACG. In the present study, we found that the peripapillary vessel density in the average, quadrant, and clock-hour sectors of PACG decreased in different degrees compared with control eyes. The inferior quadrant vessel density had the highest diagnostic value. The diagnostic abilities of vessel density in the 4 clock-hour and inferior quadrant were better than that of RNFL thickness.

In this study, the peripapillary vessel density of PACG eyes was significantly lower than that of healthy eyes, which were consistent with the studies of Rao et al. [18] and Wang et al. [19]. Rao et al. [18] used OCTA to report the diagnostic ability of peripapillary average vessel density and (divided into 6 parts) each quadrant vessel density parameters in PACG, which were similar to the diagnostic ability of RNFL thicknesses. AUCs of average peripapillary vessel density and the inferotemporal vessel density sector were 0.79 and 0.86 respectively. In the current study, the average vessel density of PACG was higher than that reported by Rao et al., while the diagnostic ability of RNFL thicknesses was similar. Our study showed that the diagnostic ability of vessel density parameters may be better than that of RNFL thicknesses in PACG. The reason may be that the severity of glaucoma in this study (VF mean deviation was − 19.4dB) was much higher than that in Rao et al. (VF mean deviation was − 9.2 dB). Previous studies showed that Peripapillary RNFL measured by OCT might not be as effective as visual field examination or macular thickness in monitoring the progression of advanced glaucoma [20, 21]. Mwanza et al. [22] ' s research reported that measuring RNFL below its minimum value would not provide useful clinical information. This result may be partly explained by that RNFL of advanced PACG change slightly, while vessel density continues to decline significantly. Previously, little was known about the diagnostic ability of clock-hour vessel density in PACG. Our study results showed that quadrant and clock-hour vascular density had good glaucoma diagnostic ability.

According to the results of the present study, we found that the vessel density at 7 o’ clock reduced most, and RNFL at 7 o’ clock also reduced most. Previous studies showed that RNFL defects were most often found in infratemporal and supratemporal sectors [23]. Anatomically, RNFL bundles are particularly thick in the superior and inferior quadrants of the optic disc. These areas are particularly vulnerable to glaucoma damage [24]. However, longitudinal study is needed to investigate the sequential relationship between vessel density reduction and RNFL atrophy. In our study, the peripapillary vessel density was closely related to the RNFL thickness and the VF mean deviation. This was consistent with the research by Zhang et al [25].

The advantage of this study was that we used imageJ software to calculate the vessel density of each clock sector that matched the clock-hour RNFL sector. We accurately matched clock-hour vessel density sectors and clock-hour RNFL sectors for the AUCs diagnostic capability comparison. However, our study had some limitations. First, PACG eyes were exposed to 0 to 3 anti-glaucoma drugs during OCTA examination, which might affect the hemodynamics of ocular blood flow and retinal vascular autoregulation [26, 27].This might further affect the measurement results of vessel density in the current study. Second, the OCTA algorithm we used included large vessels and capillaries, which might not represent the actual loss of capillaries in specific regions. Third, since it was a cross-sectional study, we couldn’t determine the cause-effect relationship between vessel density and structure. Finally, because laser iridectomy and cataract surgery might affect OCTA measurement [28], this study only included glaucoma patients whose IOP was controlled after drug treatment, and excluded those serious patients who could not be controlled by drugs, which might cause bias.

Our research showed that OCTA may provide useful information for glaucoma. The diagnostic ability of vessel density in PACG was equivalent to that of traditional RNFL measurement. When the measurement of RNFL by OCT in advanced glaucoma becomes redundant, measuring microcirculation instead of nerve tissue itself may be more meaningful in monitoring the progress of diseases. Future research is needed to evaluate the application of this new method in detecting glaucoma changes.

In summary, the diagnostic ability of vessel density in PACG is comparable to that of RNFL thickness. Understanding the spatial characteristics of Peripapillary vessel density in PACG may be helpful for clinical diagnosis and monitoring the progress of diseases.

## Data Availability

The author confirms that all relevant data are included in the article.
